# *MIR141* Expression Differentiates Hashimoto Thyroiditis from PTC and Benign Thyrocytes in Irish Archival Thyroid Tissues

**DOI:** 10.3389/fendo.2012.00102

**Published:** 2012-09-03

**Authors:** Emma R. Dorris, Paul Smyth, John J. O’Leary, Orla Sheils

**Affiliations:** ^1^Department of Histopathology, Sir Patrick Dun Research Laboratory, Trinity College Dublin, St. James’ HospitalDublin, Ireland

**Keywords:** microRNA, thyroid, Hashimoto thyroiditis, papillary thyroid carcinoma

## Abstract

MicroRNAs (miRNAs) are small non-coding RNAs approximately 22 nucleotides in length that function as regulators of gene expression. Dysregulation of miRNAs has been associated with initiation and progression of oncogenesis in humans. Our group has previously described a unique miRNA expression signature, including the MIR200 family member *MIR141*, which can differentiate papillary thyroid cancer (PTC) cell lines from a control thyroid cell line. An investigation into the expression of *MIR141* in a series of archival thyroid malignancies [*n* = 140; classic PTC (cPTC), follicular variant PTC, follicular thyroid carcinoma, Hashimoto thyroiditis (HT), or control thyrocytes] was performed. Each cohort had a minimum of 20 validated samples surgically excised within the period 1980–2009. A subset of the HT and cPTC cohorts (*n* = 3) were also analyzed for expression of *TGF*β*R1*, a key member of the TGFβ pathway and known target of *MIR141*. Laser capture microdissection was used to specifically dissect target cells from formalin-fixed paraffin-embedded archival tissue. Thyrocyte- and lymphocyte-specific markers (TSHR and LSP1, respectively), confirmed the integrity of cell populations in the HT cohort. RNA was extracted and quantitative RT-PCR was performed using comparative CT (ΔΔCT) analysis. Statistically significant (*p* < 0.05) differential expression profiles of *MIR141* were found between tissue types. HT samples displayed significant downregulation of *MIR141* compared to both cPTC and control thyrocytes. Furthermore, *TGF*β*R1* expression was detected in cPTC samples but not in HT thyrocytes. It is postulated that the downregulation of this miRNA is due, at least in part, to its involvement in regulating the TGFβ pathway. This pathway is exquisitely involved in T-cell autoimmunity and has previously been linked with HT. In conclusion, HT epithelium can be distinguished from cPTC epithelium and control epithelium based on the relative expression of *MIR141*.

## Introduction

Hashimoto thyroiditis (HT) is a chronic autoimmune disorder characterized by lymphocytic inflammation of the thyroid. This infiltration leads to apoptosis of thyrocytes and hypothyroidism. HT is the most common form of thyroiditis, with a strong prevalence for women over the age of 40 (Rosai, 2004). The triggers for this autoimmune response appear to be both humoral and cellular, with a complex etiology involving both genetic and environmental factors (Eschler et al., 2011). HT is associated with an increased incidence of papillary thyroid carcinoma (PTC). However, the relationship between HT and PTC remains controversial, with some arguing that the increased rate of screening in HT patients confounds the association between the two. However, RET/PTC chromosomal translocations, which were originally haled to be a unique biomarker of PTC, have been identified in greater than 95% of HT tissue samples, 84% of which had no evidence of malignancy (Sheils et al., 2000a). This evidence implies a shared molecular etiology between HT and PTC.

Carcinomas of the thyroid are the most common endocrine malignancy (Parkin et al., 2005, 2010). The most common thyroid carcinoma is the well-differentiated classic PTC (cPTC), which accounts for an estimated 80% of thyroid cancers. cPTC is typically easily recognized microscopically due to its characteristic papillae and optically clear nuclei. The papillae are frequently associated with the formation of follicles, the ratio of which vary greatly (Rosai, 2004). However, other subtypes of thyroid carcinoma can be more difficult to diagnose. The follicular variant of PTC (fvPTC) composes entirely or almost entirely of follicles. The diagnosis of fvPTC is primarily based on nuclear alterations associated with cPTC. Its invasiveness and incidence of nodal metastases also aid diagnoses. Follicular carcinoma of the thyroid (FTC) is usually a solitary neoplasm with a microscopic appearance varying from well-formed follicles to solid growth patterns (Rosai, 2004). Hence, accurate diagnosis of certain thyroid carcinomas, in particular the differential diagnoses of fvPTC and FTC may be problematic. Given the difficulties in differentiating between subtypes, and with a view to understanding the underlying pathology, thyroid cancer research is ongoing with a view to identifying more robust discriminators of disease and prognosis.

Profiling of microRNA (miRNA) dysregulation is a potential diagnostic tool (Braun and Hüttelmaier, 2011; Marini et al., 2011). Many miRNAs display exquisite tissue specificity that may aid in determining the tumor tissue-of-origin. It has been found that miRNA classification was more accurate than messenger RNA (mRNA) classification in determining tissue-of-origin in poorly differentiated tumors (Rosenfeld et al., 2008). An estimated 50% of miRNAs are located at genomic regions linked to cancer. Nearly every type of tumor analyzed by miRNA profiling has shown significantly altered miRNA profiles compared to normal counterparts (Calin and Croce, 2006). Investigation into miRNA deregulation in PTC has identified aberrant miRNA expression profiles in PTCs compared to normal thyroid (He et al., 2005; Cahill et al., 2006; Pallante et al., 2006, 2010; Visone et al., 2007; Aherne et al., 2008). Cahill et al. (2006) identified a miRNA signature profile for a cPTC cell line that displays significant upregulaution of *MIR141*. In the present study, an investigation into the expression of *MIR141* in a series of archival thyroid malignancies [cPTC, fvPTC, FTC, HT, or control (no evidence of disease or goiter tissues) tissues] was performed, with an aim to elucidating the expression *MIR141* across subtypes of follicular thyroid neoplasia. Each cohort had a minimum of 20 validated samples surgically excised within the period 1980–2009. Multiple members of the TGFβ pathway, including transforming growth factor, beta receptor 1 (TGFβR1), have been identified to be direct targets of MIR141 (Braun et al., 2010; Senanayake et al., 2012). This pathway is involved in signaling for the cytokine IL-2, which has a well documented association with HT (Weijl et al., 1993). Hence a subset of the cPTC and HT cohort were analyzed for *TGF*β*R1* gene expression.

## Materials and Methods

### Ethical approval

Full ethical approval was obtained for this study from the Joint St. James’s Hospital/Adelaide and Meath National Children’s Hospital Research Ethics Committee.

### Tissue sample

All samples chosen for this analysis were from different thyroid tissues excised between 1980 and 2009. All samples were derived from an Irish population to limit geographical variation. A cohort of 140 archival cases of formalin-fixed paraffin-embedded (FFPE) tissues was obtained from the archives at St. James’s Hospital. The cohort comprised of 30 control (no evidence of malignancy or goiter) thyroid tissues, 30 cPTC tissues, 28 fvPTC tissues, 26 FTC tissues, and 26 HT tissues. All tissues had been assessed by a registered histopathologist and diagnoses confirmed according to the World Health Organisation classification guidelines.

### Laser capture microdissection

The FFPE tissue were cut using a HM325 rotary microtome (MSC, Dublin, Ireland) to 7 μm, mounted on uncharged slides and dried on a heating block to remove excess moisture. Slides were dewaxed and hematoxylin and eosin stained using the automated Tissue-Tek DRS 2000 Autostainer (Sakura, CA, USA). Stained slides were mounted onto an Arcturus^xt^ Microdissection instrument (MDS Analytical Technologies, CA, USA). LCM was used to capture (infrared 810 nm) specific cell populations of interest onto CapSure^®^ Macro LCM caps (MDS Analytical Technologies, CA, USA). To confirm the purity of thyrocytes captured from HT tissues, an area containing lymphocytic infiltrate from the same 7 μm section of a representative subset of the HT tissues were also microdissected on to separate caps for real-time PCR comparison of thyrocyte-specific and lymphocyte-specific markers. Following microdissection the transfer film containing the captured cells was peeled off the CapSure^®^ macro LCM cap and placed into sterile DNA LoBind tubes (Eppendorf AG, Hamburg, Germany) to maximize downstream recovery.

### Nucleic acid extraction

RNA was extracted using the RecoverAll™ total nucleic acid isolation kit optimized for FFPE samples (Applied Biosystems, CA, USA) following the supplied recommended protocol, with step B (deparaffinization) omitted as the sections had been deparaffinized during the H&E staining protocol. The RNA quantity and quality were assessed using the NanoDrop^®^ ND-1000 Spectrophotometer (Wilmington, USA).

### miRNA analysis

Inventoried TaqMan^®^ miRNA Assay (Applied Biosystems, CA, USA) were used to synthesize single stranded MIR141 and RNU6B (endogenous control) cDNA according to the supplied TaqMan^®^ miRNA assays protocol. Reverse transcription was performed on a Gene Amp PCR System 9600 (Perkin Elmer, MA, USA) with thermal cycling parameters set as per the TaqMan^®^ miRNA assays protocol.

Real-time PCR was performed according to TaqMan^®^ miRNA assays protocol using MIR141-specific or RNU6B-specific inventoried real-time miRNA expression assays. The recommended protocol was adjusted such that the RT product was diluted 1/5 for use in the assay, with the nuclease-free water adjusted accordingly. Real-time PCR was performed on an ABI Prism 7900HT Sequence Detection System (Applied Biosystems, CA, USA) according to supplied recommended protocol.

### mRNA analysis

Reverse transcription was performed using the High Capacity cDNA Reverse Transcription Kit (Applied Biosystems, CA, USA) as per manufacturers protocol. Real-time PCR was performed using inventoried TaqMan^®^ Gene Expression Assays according to manufacturer supplied protocol for thyroid stimulating hormone receptor (TSHR), lymphocyte-specific protein 1 (LSP1), TGFβR1, and the endogenous control glyceraldehydes-3 phosphate dehydrogenase (GAPDH).

### Data analysis

MicroRNAs and gene expression was measured by relative quantitation (RQ). The comparative *C*_T_ (ΔΔ*C*_T_) method was used to calculate RQ of mRNA and miRNA expression, using GAPDH and RNU6B as the endogenous control for mRNA and miRNA expression, respectively. The same calibrator and threshold levels were used for all samples. Comparative studies were preformed on the data using the SDS2.1 software (Applied Biosystems, CA, USA). The study was then exported to the Microsoft^®^ Office Excel^®^ 2007 software (Microsoft Corporation, WA, USA), Minitab 16^®^ (Minitab Inc., USA) and to STATA 8 software (StataCorp LP, TX, USA) for further analysis. Differentially expressed miRNA patterns across tissue types were identified by means of a one-way ANOVA. Statistical significance was set at *p* < 0.05. The relative expression (RQ) of TSHR and LSP1 in LCM captured isolated thyrocytes was compared to that of parallel LCM captured populations containing lymphocytic infiltration. Statistical significance was set at (±) twofold expression of thyrocyte-specific populations compared to parallel populations containing lymphocytic infiltrate.

## Results

### Laser capture microdissection

Of the 140 thyroid tissues in the cohort, laser capture microdissection of 24 tissues did not capture either sufficient quantity or sufficient quality of cells to extract detectable levels of RNA. Of the remaining 116 tissues, 111 (95.69%) expressed the endogenous control, *RNU6B*, which was selected as the internal normalization control for miRNA quantitation. Of the 111 samples expressing the endogenous control, 104 samples also expressed *MIR141*. This cohort of samples expressing both *RNU6B* and *MIR141* consisted of 20 cPTC samples, 20 fvPTC samples, 19 FTC samples, 21 HT samples, and 24 control samples.

### Characterization of mRNA from HT samples

**Figure 1:**
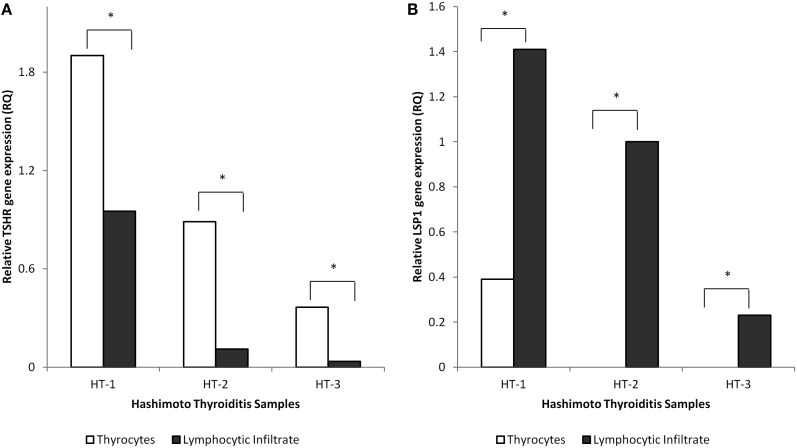
**Characterization of isolated thyrocytes from representative HT tissues**. The thyrocyte population was captured using LCM. An adjacent/interspersed area from the same 7 μm section containing lymphocytic infiltrate was captured on a separate cap for comparison of gene expression. Relative gene expression of *TSHR* **(A)** and *LSP1* **(B)** was measured between groups. The isolated thyrocytes had significantly upregulated levels of *TSHR* compared to populations containing lymphocytic infiltrate. *LSP1* was only detected in 1 sample (HT-1) of isolated thyrocytes and was significantly downregulated compared to the parallel lymphocyte sample. *Indicates statistical significance of ± twofold difference in relative gene expression of thyrocyte-specific populations compared to parallel populations containing lymphocytic infiltrate. *Y*-axis is fold change in gene expression (RQ).

To confirm the purity of the LCM harvested cell populations (thyrocyte and lymphocyte), HT tissues were assayed for thyrocyte- and lymphocyte-specific markers (*TSHR* and *LSP1*, respectively). mRNA extracted from thyrocytes harvested from HT tissues was compared to mRNA extracted from interspersed/adjacent areas of lymphocytic infiltrate captured separately but from the same 7 μm sections. As expected, *THSR* was significantly upregulated in each isolated thyrocyte population compared to each parallel sample containing lymphocytic infiltrate. Conversely, *LSP1* was detected in all of the harvested lymphocyte samples but was only detected in a single thyrocyte sample, albeit displaying significant downregulation compared to its paired lymphocyte extract (Figure 1).

### Expression of *MIR141* in thyroid tissues

**Figure 2:**
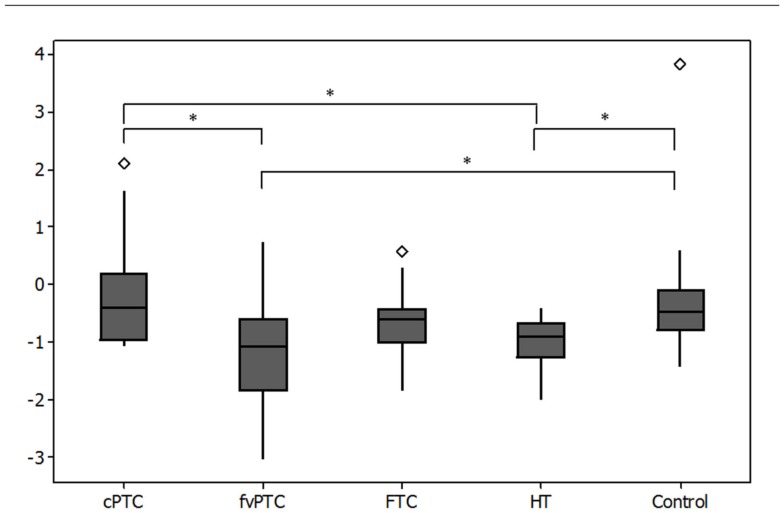
**Boxplot of relative quantitation (RQ) of change in expression of *MIR141* across cohorts of follicular thyroid malignancy**. The expression of *MIR141* is statistically significantly downregulated in HT tissues compared to both control and cPTC tissues. The fvPTC tissues also display a statistically significant downregulation of *MIR141* in comparison to both control and cPTC tissues. *Y*-axis is log(RQ). *Indicates statistical significance (*p* < 0.05) as identified by ANOVA, ◊ indicates outliers.

A comparison of relative gene expression across the thyroid cohorts identifies an association between *MIR141* expression and malignant thyroid phenotypes. Analysis of variance (one-way ANOVA) was performed on log transformed data between cohorts and identified statistical significance between populations (*p* = 0.01, *F* = 5.40). Residual analysis on log transformed data confirmed normally distributed populations for each cohort (*p* < 0.005). Expression of *MIR141* was significantly downregulated (*p* < 0.05) in HT samples compared to both control and cPTC samples (Figure 2). Analysis of variance also demonstrates that the follicular variant of PTC displays a statistically significant (*p* < 0.05) pattern of downregulation of *MIR141* expression compared to both control and cPTC samples.

### *TGFBR1* is expressed in cPTC but not in HT thyrocytes

A subset of HT and cPTC samples (*n* = 3/cohort) were assayed for the expression of *TGF*β*R1*, a member of the TGFβ pathway and known target of MIR141 (Braun et al., 2010). Expression of *TGF*β*R1* was detected in the all of cPTC samples but not in HT thyrocytes. Paired HT lymphocytes (*n* = 3) were also assayed for *TGF*β*R1* and expression was detectable in these samples. As such, the cPTC subset (*n* = 3) were assayed for *LSP1* gene expression to confirm that the *TGF*β*R1* expression detected was not due to lymphocyte contamination. *LSP1* gene expression was not detectable in the cPTC samples.

## Discussion

Inflammation and thyroid cancer are strongly linked. PTCs have a penchant to attract a florid of lymphocytic infiltrate. However, they can persist and metastasize in the presence of this infiltrate. Normal inflammation is self-limiting because the production of anti-inflammatory and pro-inflammatory cytokines is tightly regulated. During normal inflammation, blocking apoptosis provides time for DNA repair and the production of reactive oxygen species (ROS) fights against viral threat. However, the failure of mechanisms required for resolving the inflammatory response or the persistence of the initiating factors can result in chronic inflammation. ROS are mutagens. The blocking of apoptosis in cells that are gaining oncogenic mutations can lead to tumor progression. Thus chronic inflammation can act as both an initiator (DNA damage and growth signals) and as a promoter (blocking apoptosis and stimulating cell proliferation), which over time can induce oncogenesis.

Formalin-fixed paraffin-embedded tissue samples were used in this study. While FFPE preserves tissue for morphological analysis, the effect of formalin fixation on nucleic acids can hamper molecular analysis as nucleic acids become cross-linked during fixation. There are no standardized guidelines for processing FFPE tissues (Blow, 2007), and as such there can be great variation in the time between ligation of circulation and fixation. Nucleic acids suffer damage through fixation. The greater the time prior to fixation, the greater the exposure to nucleases and modifying enzymes, which are upregulated during ischemia. Nucleic acids are often irreversibly damaged and can become increasingly fragmented during prolonged storage (Burgemeister, 2011). Mature miRNAs, however, are approximately 22 nucleotides in length. Due to this small size they tend not to suffer from the fragmentation effect. Thus, FFPE tissues tend to yield miRNA more effectively than mRNA. It has previously been demonstrated that FFPE thyroid tissues are a suitable resource for miRNA expression analysis (Tetzlaff et al., 2007; Chen et al., 2008; Sheu et al., 2010). The method used for miRNA extraction in this study has previously been demonstrated to yield miRNA with expression levels that positively correlate with expression levels from snap frozen tissue (Li et al., 2007).

MicroRNAs have emerged as key regulators of the immune system, displaying unique expression profiles in cells of the adaptive and innate immune system (O’Connell et al., 2010). Aberrant expression of miRNAs has been documented in a range of diseases associated with the immune system, including cancer and autoimmunity (Calin et al., 2002; Xiao et al., 2008). MiRNA signatures that can distinguish thyroid malignancies in fine needle aspiration, FFPE, and fresh surgical samples have been identified (He et al., 2005; Pallante et al., 2006; Tetzlaff et al., 2007; Chen et al., 2008; Nikiforova et al., 2008; Sheu et al., 2010; Shen et al., 2012; Vriens et al., 2012). It has been demonstrated that *MIR141* is upregulated in a cell line model of ret/PTC-1 associated PTC (Cahill et al., 2006). In the current study, we investigated if the pattern of *MIR141* expression would translate to clinical specimens. In fact, the pattern of upregulated *MIR141*was not observed in the study sample. This may be due to (a) variation between *in vitro* and *in vivo* expression or (b) the low rate of ret/PTC variants in the samples analyzed (Table 1).

**Table 1:** **Mutational status of *RET/PTC-1* and *RET/PTC*-3 for PTC samples expressing *MIR141***.

PTC variant	Year of biopsy	RET/PTC mutation	PTC variant	Year of biopsy	RET/PTC mutation
Classic	1980	Unknown	Follicular variant	1986	Negative
Classic	1985	Unknown	Follicular variant	1987	Unknown
Classic	1987	Unknown	Follicular variant	1989	Negative
Classic	1994	RET/PTC-1	Follicular variant	1991	Negative
Classic	1998	Negative	Follicular variant	1991	Negative
Classic	1998	RET/PTC-1	Follicular variant	1992	RET/PTC-1
Classic	2002	Negative	Follicular variant	1992	Unknown
Classic	2002	Negative	Follicular variant	1992	Unknown
Classic	2002	Negative	Follicular variant	1992	Negative
Classic	2003	Negative	Follicular variant	1993	RET/PTC-3
Classic	2004	Negative	Follicular variant	1996	Unknown
Classic	2004	Negative	Follicular variant	2001	Negative
Classic	2004	Negative	Follicular variant	2003	Negative
Classic	2004	Negative	Follicular variant	2003	Negative
Classic	2004	Negative	Follicular variant	2004	Negative
Classic	2004	Negative	Follicular variant	2004	Negative
Classic	2005	Negative	Follicular variant	2004	Negative
Classic	2005	Negative	Follicular variant	2004	Negative
Classic	2005	Negative	Follicular variant	2005	Negative
Classic	2005	Negative	Follicular variant	2005	Negative

In this study a significantly decreased expression of *MIR141* was identified in thyrocytes isolated from HT tissues compared to both control and cPTC samples. This is of particular interest as it has been postulated that HT represents a circumstance in which thyrocytes are in a state between normal and neoplastic (Sheils et al., 2000b). Histologically, HT can present with the occurrence of nuclear clearing and overlapping cells associated with PTC, but without evidence of malignancy (Rosai, 2004). On a molecular level, previous studies (Wirtschafter et al., 1997; Sheils et al., 2000a; Rhoden et al., 2006) detected that up to 95% of HT samples displayed evidence of ret/PTC-1 activation, including HT samples that had no evidence of malignancy. The ret/PTC chromosomal rearrangement was previously considered to be a hallmark of PTC. However, evidence of their presence in HT samples indicates a more complex role of this genetic alteration. Furthermore; the occurrence of non-specific lymphocytic infiltrate in PTC tissues has been positively associated with the presence of RET/PTC-1 expression (Sheils et al., 2000b). This has lead to the postulation that antigenic alterations induced by genetic alterations such as the activation of c-ret are responsible for eliciting an immune response within the thyroid tissue. In HT, it is feasible that the presence of genetic alterations such as c-ret elicit an immune response to curtail the development of PTC. A subset of patients may be successfully in this regard but with background damage of autoimmune destruction of thyroid tissue. However, the relationship between HT and PTC remains a highly controversial issue.

MIR141 has been confirmed to directly target a number of genes. Multiple members of the TGFβ pathway have been identified to be direct targets of MIR141 (Braun et al., 2010; Senanayake et al., 2012). Braun et al. (2010) demonstrated direct targeting of TGFβR1 and SMAD family member 2 (SMAD 2). Both TGFβR1 and SMAD2 are negatively regulated by MIR141. These key members of the TGFβ signaling pathway act to signal for interleukin-2 (IL-2), a cytokine involved in T-cell proliferation. T-cells play a crucial role in the development of HT as they secrete inflammatory cytokines in reaction to auto-antigens; primarily TSHR and thyroid peroxidase (TPO). IL-2 is essential for T-cell determination of self from non-self. IL-2Rβ/IL-2 knock-out mice developed a marked increase in T-cell mediated autoimmunity and deregulated T-cell proliferation. Furthermore, IL-2 is used as treatment for certain cancers, which is documented to result in an increased risk of HT (Weijl et al., 1993). Thus, the association of MIR141 with the regulation of this pathway may be reflected in its downregulation in HT epithelium. The relative expression of *TGF*β*R1* was assayed in a subset of samples (*n* = 3) in this study. *TGF*β*R1* was found to be expressed in cPTC samples but not in isolated HT thyrocytes. *MIR141* expression is upregulated in these cPTC samples compared to the isolated HT thyrocyte samples. This initial data supports the argument for further investigation into the association of MIR141, the TGFβ signaling pathway and HT.

Thyroid carcinomas frequently present with multiple phenotypic tumor-foci within a single thyroid gland. Direct experimental analysis of these tissues will therefore reflect the predominant cell type within the tissue which may not be the cell of interest nor be biologically relevant to the neoplasm under investigation. MiRNA profiles have been found to distinguish tumor types within a single thyroid biopsy (Aherne et al., 2008). This highlights the requirement to extract a pure population of the specific tumor cells in order to identify and accurately define the molecular processes in pathogenic lesions. LCM provides a non-damaging microdissection process which allows isolation of pure populations while preserving the integrity of the captured material (Liu, 2010). In this study, LCM was utilized to specifically select the cell-types of interest and therefore reduce the confounding factors associated with whole section analysis. This enforces the accuracy of the associations identified between MIR141 and malignant thyroid phenotypes.

In conclusion, HT epithelium can be distinguished from neoplastic follicular thyroid epithelium isolated from cPTC and control epithelium based on the relative expression of *MIR141*. It is postulated that the downregulation of this miRNA may be due, at least in part, to its involvement in regulating the TGFβ signaling pathway. The MIR200 family, including MIR141, have been demonstrated to directly regulate members of this pathway as part of a regulatory feedback loop. The preliminary data presented here in combination with data from the literature (Braun et al., 2010; Senanayake et al., 2012) leads this postulation to warrant further investigation. The TGFβ pathway is exquisitely involved in T-cell autoimmunity and has been linked with HT for some time, as treatment of cancer patients with IL-2, a downstream target of the pathway, resulted in increased incidence of HT (Weijl et al., 1993). Further investigation of MIR141 and its regulatory networks may aid in elucidating the molecular mechanisms that result in the fundamental physiological differences between these malignant thyroid phenotypes.

## Conflict of Interest Statement

The authors declare that the research was conducted in the absence of any commercial or financial relationships that could be construed as a potential conflict of interest.
